# Expression of Core Hippo Pathway Proteins in Cervical Cancer and Their Association with Clinicopathologic Parameters

**DOI:** 10.3390/medicina61122134

**Published:** 2025-11-29

**Authors:** Min Hye Kim, Juseok Yang, Dae Hyun Song, Cho Hee Kim, Jeong Kyu Shin, Won Jun Choi, Jong Chul Baek

**Affiliations:** 1 Department of Pathology, Institute of Medical Science, School of Medicine, Gyeongsang National University, Jinju 52727, Republic of Korea; joymine86@naver.com (M.H.K.); golgy@hanmail.net (D.H.S.); chohing9@gmail.com (C.H.K.); 2 Department of Pathology, Gyeongsang National University Hospital, Jinju 52727, Republic of Korea; 3 Department of Obstetrics and Gynecology, Institute of Medical Science, School of Medicine, Gyeongsang National University, Jinju 52727, Republic of Korea; yangandshin@gmail.com (J.Y.); 2848049@hanmail.net (J.K.S.); choiwj@gnu.ac.kr (W.J.C.); 4 Department of Obstetrics and Gynecology, Gyeongsang National University Changwon Hospital, Changwon 51472, Republic of Korea; 5 Department of Pathology, Gyeongsang National University Changwon Hospital, Changwon 51472, Republic of Korea; 6 Department of Obstetrics and Gynecology, Gyeongsang National University Hospital, Jinju 52727, Republic of Korea

**Keywords:** cervical cancer, hippo pathway, YAP, p-YAP, MST1, LATS1, prognosis, siRNA knockdown, tumor migration

## Abstract

***Background:*** The Hippo signaling pathway, a highly conserved regulatory cascade, regulates tissue homeostasis, organ size, and tumor suppression. Dysregulation of this pathway contributes to oncogenesis in various human malignancies; however, its clinicopathologic relevance in cervical cancer has not been completely elucidated. Therefore, this study aimed to investigate the expression patterns of key Hippo pathway proteins and analyze their associations with tumor behavior and clinicopathologic features in cervical carcinoma. ***Materials and Methods:*** Ninety-nine cervical cancer specimens obtained from hysterectomies performed at Gyeongsang National University Hospital (2012–2019) were retrospectively analyzed. Immunohistochemical staining for Yes-associated protein (YAP), phosphorylated YAP (p-YAP), mammalian sterile-20-like kinase-1 (MST1), and large tumor suppressor kinase-1 (LATS1) was performed on tissue microarrays. Chi-square or Fisher’s exact tests and logistic regression were employed for assessing associations between marker expression and clinicopathologic variables. Functional validation was conducted via small interfering RNA-mediated YAP knockdown in Caski cervical cancer cells, with reverse transcription-polymerase chain reaction, Western blotting, and wound-healing assays assessing YAP suppression and cell migration. ***Results:*** YAP and p-YAP were expressed in 71.8% and 62.6% of tumors, respectively; MST1 in 82.8%; and LATS1 in 22.2%. YAP and p-YAP overexpression was correlated with larger tumor size (*p* = 0.013 and *p* = 0.011) and higher International Federation of Gynecology and Obstetrics stage (*p* = 0.007 and *p* < 0.001). YAP and p-YAP expression was positively correlated (odds ratio, 4.34; 95% confidence interval, 1.70–11.61). MST1 or LATS1 expression demonstrated no significant associations. In vitro, YAP silencing decreased mRNA and protein expression levels and significantly impaired cell migration, supporting its role in tumor aggressiveness. ***Conclusions:*** YAP and p-YAP overexpression are associated with advanced stage and larger tumor size in cervical cancer, indicating Hippo pathway dysregulation. YAP functional suppression attenuated migratory capacity, highlighting YAP as a promising prognostic biomarker and therapeutic target.

## 1. Introduction

The Hippo signaling pathway, initially discovered in Drosophila as a tumor suppressor [1,2,3,4], is highly conserved across vertebrates and is significantly involved in tissue growth, cell proliferation, homeostasis, and angiogenesis [5,6,7,8]. At its core, a kinase cascade involving mammalian sterile-20-like kinase-1/2 (MST1/2) and large tumor suppressor kinase-1/2 (LATS1/2) phosphorylates the downstream effectors Yes-associated protein (YAP) and WW-domain-containing transcription regulator protein 1 (also known as transcriptional co-activator with PDZ-binding motif [TAZ]) [6,7,9,10]. YAP and TAZ phosphorylation causes their sequestration in the cytoplasm and subsequent proteasome-induced degradation. Only the dephosphorylated forms of YAP and TAZ can translocate into the nucleus, where they bind to TEA domain family transcription factors (TEAD1–4) [11,12] to activate target gene expression, including connective tissue growth factor (CTGF), cysteine-rich angiogenic inducer 61 (CYR61), and AXL receptor tyrosine kinase (AXL), thereby supporting cell proliferation and survival [13,14,15].

Aberrant Hippo signaling pathway regulation has been implicated in the pathogenesis of various human malignancies [16,17,18], including gynecologic cancers [19,20,21]. YAP and TAZ nuclear overexpression has been linked to poor clinical outcomes and therapeutic resistance [22], whereas modulation of this pathway has become a promising strategy for immune checkpoint therapy [16,17,23,24].

Globally, cervical cancer remains the fourth most common malignancy among females, posing a particularly high mortality burden in low- and middle-income countries [25]. Prognosis for invasive cervical cancer remains poor despite the availability of prophylactic human papillomavirus (HPV) vaccination. Although recent studies have suggested an association between the Hippo pathway and cervical cancer progression [26,27,28], drug resistance [29], and HPV-related mechanisms [19], systematic evaluation of Hippo core protein expression with respect to clinicopathologic features of cervical cancer remains lacking.

Therefore, this study aimed to investigate the expression patterns of key Hippo pathway proteins (YAP, p-YAP, MST1, and LATS1) in cervical cancer and to evaluate their associations with clinicopathologic parameters. To complement the histopathologic analysis, functional experiments were performed using small interfering RNA (siRNA)–mediated YAP knockdown in CaSki cervical cancer cells to assess the effects on mRNA and protein expression as well as cellular migration. This study uniquely integrates a dual investigative approach—comprehensive immunohistochemical evaluation of archival human tissues and in vitro functional validation—to provide a more biologically grounded understanding of Hippo pathway dysregulation in cervical carcinogenesis. Through this combined design, we sought to clarify the role of YAP in tumor progression and explore its utility as a prognostic biomarker and potential therapeutic target.

## 2. Materials and Methods

### 2.1. Sample Collection

Surgical specimens from patients who underwent hysterectomy and were diagnosed with cervical squamous cell carcinoma (SCC) were retrospectively collected. Overall, 99 patients who underwent either laparoscopy or laparotomy at Gyeongsang National University Hospital (GNUH; Jinju, South Korea) from January 2012 to December 2019 were included. Laparotomy, conventional laparoscopy, and robot-assisted laparoscopy were surgical approaches employed, and the type of hysterectomy was determined according to the clinical stage of cervical cancer. Relevant clinicopathologic data, including patient age, parity, HPV status, recent Papanicolaou smear results, serum albumin levels, SCC antigen levels, FIGO stage, tumor size, and depth of stromal invasion, were collected from electronic medical records. Tumor staging was performed according to the Seventh Edition of the American Joint Committee on Cancer and the Fourth Edition of the World Health Organization classification.

### 2.2. Tissue Microarray (TMA) Construction and Ethical Approval

Surgically resected specimens were fixed overnight in 20% buffered neutral formalin and subsequently embedded in paraffin. Hematoxylin and eosin-stained slides were prepared from the paraffin-embedded blocks. Representative sections from the prepared slides were reviewed, and two areas best representing the tumor were selected and marked. From each selected area, 3 mm tissue cores were obtained and transplanted into a recipient TMA block.

The Institutional Review Board of GNUH approved the study protocol (IRB approval number: 2020-04-006). Owing to the retrospective design of this study and the use of anonymized archival materials, the requirement for informed consent was waived. All procedures were performed following the principles of the Declaration of Helsinki.

### 2.3. Immunohistochemical (IHC) Staining

IHC staining was performed on 4-μm-thick sections prepared from TMA blocks. Four primary antibodies, including phosphorylated YAP (p-YAP), YAP, MST1, and LATS1, were used for staining following the manufacturers’ protocols. The following antibodies were utilized: p-YAP (Ser127) (1:1000, monoclonal, clone D9W2I, #13008; Cell Signaling Technology, Danvers, MA, USA), YAP (1:200, monoclonal, clone D8H1X, #14074; Cell Signaling Technology, Danvers, MA, USA), MST1 (1:250, polyclonal, #ab87322; Abcam, Cambridge, MA, USA), and LATS1 (1:200, polyclonal, #PA5-115498; Invitrogen, Carlsbad, CA, USA). All staining procedures were performed using an automated immune-stainer (Benchmark Ultra, Ventana Medical Systems Inc., Tucson, AZ, USA).

### 2.4. Immunostaining Evaluation

**Figure 1:**
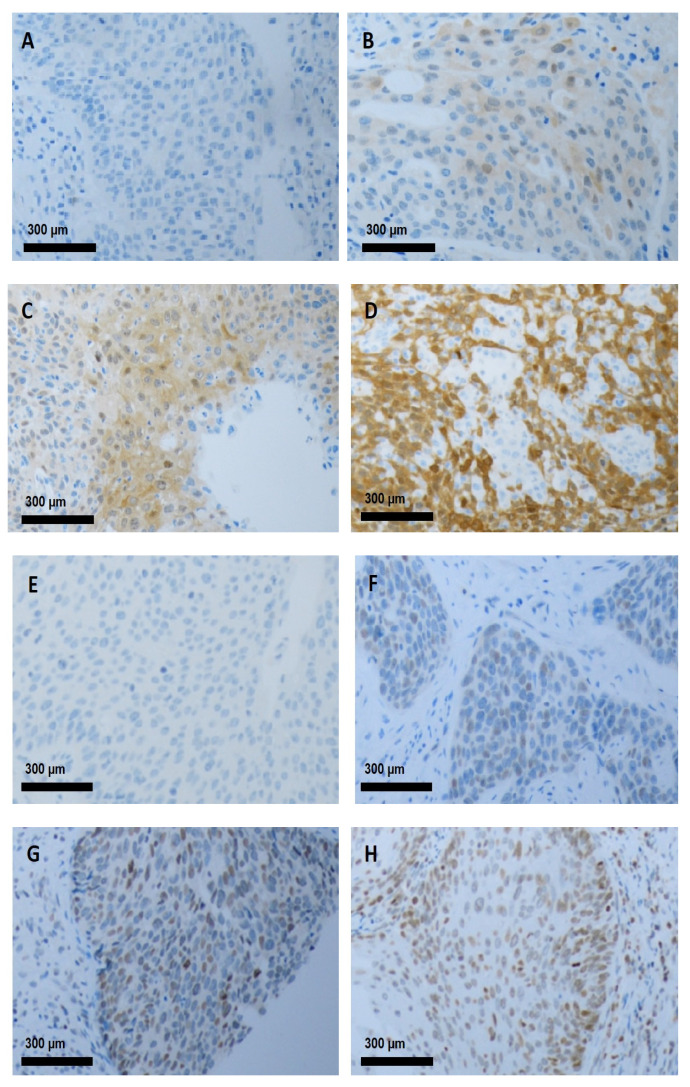
Representative images of immunohistochemical staining for phosphorylated YAP (p-YAP) and large tumor suppressor kinase-1 LATS1 in cervical cancer tissue specimens. (**A**–**D**) p-YAP cytoplasmic expression is scored on a four-point intensity scale: (**A**) 0 (absent), (**B**) 1+ (mild), (**C**) 2+ (moderate), and (**D**) 3+ (strong). (**E**,**H**) LATS1 nuclear expression is scored on a four-point intensity scale: (**E**) 0 (absent), (**F**) 1+ (mild), (**G**) 2+ (moderate), and (**H**) 3+ (strong). All images are taken at an original magnification of 100×.

Immunoreactivity was semi-quantitatively evaluated on the basis of staining intensity and subcellular localization. YAP, p-YAP, and MST1 demonstrated predominantly cytoplasmic staining patterns, whereas LATS1 was largely observed as a nuclear signal (Figure 1). Each marker was assessed using a four-point scale reflecting staining intensity (0 = absent, 1+ = mild, 2+ = moderate, and 3+ = strong). To ensure consistency with previous studies, approximate thresholds corresponding to the proportion of positive tumor cells were referenced: <1% (absent), 1–10% (mild), 10–50% (moderate), and ≥50% (strong). In cases of diffuse homogeneous staining, the final score reflected the overall staining intensity rather than proportion alone (e.g., diffuse weak staining was classified as score 1). Two pathologists blinded to clinicopathologic data independently evaluated all slides, and discrepancies were resolved by consensus. For analytical purposes, tumors with staining intensities of 0 or 1+ were classified as low expression, whereas those with 2+ or 3+ intensities were considered high expression. This two-tier cut-off follows the semiquantitative system used in our previous study on Hippo pathway markers and is consistent with IHC-based scoring methods applied in other Hippo-pathway investigations [21].

### 2.5. Cell Culture and siRNA-Mediated YAP Knockdown

Caski human cervical carcinoma cells were obtained from the Korean Cell Line Bank and cultured in RPMI-1640 medium (Gibco, Thermo Fisher Scientific, Waltham, MA, USA) supplemented with 10% fetal bovine serum and 1% penicillin–streptomycin at 37 °C in a humidified atmosphere containing 5% CO_2_. For transient YAP suppression, cells were transfected with YAP-specific siRNA or negative control siRNA (Bioneer, Daejeon, Republic of Korea) using Lipofectamine RNAiMAX (Invitrogen, Carlsbad, CA, USA) following the manufacturer’s protocol. Final siRNA concentrations of 25, 50, and 100 nM were tested, and cells were harvested at 48 h post-transfection for subsequent analyses. Total RNA was extracted using TRIzol reagent (Invitrogen), and complementary DNA was synthesized using a PrimeScript RT kit (Takara, Japan). Polymerase chain reaction (PCR) amplification was performed under optimized conditions (58 °C annealing, 37 cycles), using GAPDH as an internal control. For protein analysis, total cell lysates were prepared using RIPA buffer, separated by sodium dodecyl sulfate–polyacrylamide gel electrophoresis, and transferred onto PVDF membranes. The membranes were probed with primary antibodies against YAP, p-YAP, and GAPDH (Cell Signaling Technology), followed by HRP-conjugated secondary antibodies. Bands were visualized using an ECL detection system, and densitometric analysis was performed using ImageJ software (version 1.53; National Institutes of Health, Bethesda, MD, USA). A wound-healing assay was performed for assessing the functional impact of YAP knockdown. Following siRNA transfection (100 nM), a straight scratch was created on confluent monolayers using a sterile 200 µL pipette tip. Cells were washed with PBS to remove debris and cultured in a serum-free medium. Wound closure was monitored at 0, 12, 24, and 48 h under an inverted microscope, and the wound area was measured in three randomly selected fields per sample.

### 2.6. Statistical Analysis

Statistical analyses were performed using appropriate comparative methods based on variable type. Categorical variables, including IHC expression levels, were analyzed using the chi-square test or Fisher’s exact test. Univariate logistic regression was used to evaluate associations between Hippo pathway markers and clinicopathologic parameters. Multivariable regression was not performed due to substantial missing data for key covariates—particularly HPV status and serum SCC antigen levels—which precluded reliable adjustment for confounders. Quantitative assay data were expressed as mean ± standard deviation (SD) and compared using Student’s *t*-test. A two-sided *p* < 0.05 was considered statistically significant. Quantitative measurements from functional assays were expressed as mean ± standard deviation (SD) and compared using Student’s *t*-tests. Statistical significance was defined as a two-sided *p* < 0.05. All statistical analyses were performed using Statistical Package for the Social Sciences (version 24.0; IBM Corp., Armonk, NY, USA) and R software (version 4.51; R Project for Statistical Computing, Vienna, Austria).

## 3. Results

### 3.1. Clinicopathologic Features of the Patients

The clinicopathologic characteristics of the cervical cancer cohort are summarized in Table 1. The mean age of the patients at hysterectomy was 52.6 years. The high-parity group, defined as parity ≥ 3, accounted for more than one-third of the cohort (39.4%). Less than 40% of the patients were positive for high-risk HPV genotypes (16, 18, 31, 33, 51, 52, 56, and 58), as approximately 50% of the cohort had unknown HPV status. The mean SCC antigen serum level was 4.73 (range, 0.5–40.8) ng/mL. Most patients presented with early FIGO stages, with 16.2% (*n* = 16) and 50.0% (*n* = 50) in stages IA and IB, respectively. More than two-thirds of the patients underwent laparoscopic surgery, and modified radical hysterectomy (*n* = 63, 63.6%) was more frequently performed than radical hysterectomy (*n* = 36, 36.4%).

### 3.2. IHC Staining in Cervical Cancer

The expression of Hippo pathway core proteins in cervical cancer is presented in Table 2. YAP expression was observed in 71 cases (71.8%), whereas p-YAP exhibited a slightly lower expression rate in 62 cases (62.6%). MST1 was expressed in 82 cases (82.8%), whereas LATS1 was positive in only 22 cases (22.2%). No TAZ expression was detected in this cohort.

### 3.3. Association Between Clinicopathologic Features and Protein Expression

The association between clinicopathologic variables and YAP, p-YAP, MST1, and LATS1 expression is summarized in Table 3. FIGO stage and tumor size were significantly associated with the expression of YAP (*p* = 0.007 and *p* = 0.013, respectively) and p-YAP (*p* < 0.001 and *p* = 0.011, respectively). HPV status and Pap smear subgroup analysis results were unreliable owing to the high proportion of missing data. More than 50% of the tumors with stromal invasion exhibited lower p-YAP expression; however, this did not reach statistical significance (*p* = 0.051). High serum SCC antigen level, high parity, lymphovascular invasion, and lymph node metastasis revealed no significant association with Hippo core protein expression. LATS1 expression demonstrated no significant association with any clinicopathologic variables.

### 3.4. Associations Among Hippo Core Protein Expression

The associations among Hippo pathway core protein expression are shown in Table 4. TAZ expression was absent in this cohort; therefore, analyses were restricted to YAP, p-YAP, MST1, and LATS1. YAP and p-YAP expression were positively associated, and univariable logistic regression revealed that the OR for concordant YAP and p-YAP expression was 4.342 (95% confidence interval, 1.695–11.614). In contrast, MST1 and LATS1 expression revealed no significant association with YAP (*p* = 0.520 and *p* = 0.172, respectively) or p-YAP expression (*p* = 0.569 and *p* = 0.454, respectively).

### 3.5. Functional Validation of YAP Knockdown in Caski Cervical Cancer Cells

Cell-based functional assays were performed in Caski cells following siRNA-mediated YAP knockdown to confirm the biological role of YAP and the association between Hippo pathway activity and cervical cancer aggressiveness. These assays encompassed mRNA quantification by PCR, protein-level verification by Western blotting, and a wound-healing assay for cell migration assessment.

#### 3.5.1. YAP Knockdown Efficiency at the mRNA Level

**Figure 2:**
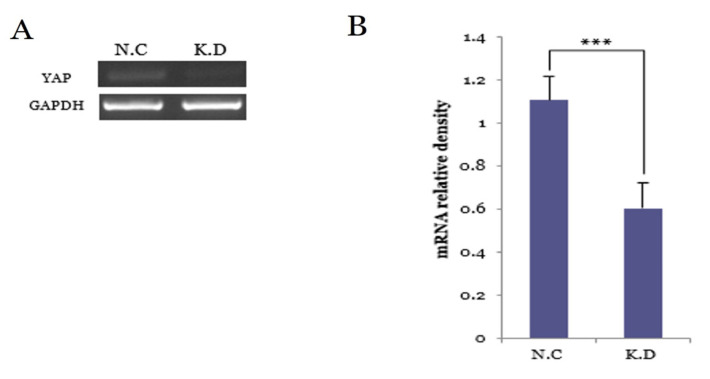
YAP knockdown efficiency confirmed by RT-PCR in Caski cervical cancer cells. (**A**) Representative RT-PCR images from four independent experiments (#1–#4), demonstrating consistently reduced YAP mRNA expression in YAP-silenced (K.D) cells compared with negative control (N.C) cells. GAPDH served as the internal control and exhibited stable expression. Full-length, uncropped gels are provided in Supplementary Figure S1. (**B**) Relative YAP mRNA expression normalized to GAPDH, based on densitometric analysis using ImageJ. Data represent the mean ± SD from four independent experiments. YAP expression decreased by approximately 50% in the K.D group (*** *p* < 0.001).

Reverse transcription-PCR analysis confirmed effective silencing of YAP at the transcriptional level in Caski cells (Figure 2). Under optimized amplification conditions (58 °C, 37 cycles), all four independent experiments consistently showed decreased YAP band intensity in the siRNA-treated knockdown (K.D) group, while GAPDH expression remained stable, indicating uniform RNA loading. Densitometric quantification using ImageJ demonstrated an approximately 50% reduction in YAP mRNA levels in the K.D group relative to the negative control (N.C) (*** *p* < 0.001). These findings verify successful YAP knockdown and support progression to subsequent protein-level and functional assays.

#### 3.5.2. YAP and P-YAP Suppression by SiRNA

**Figure 3:**
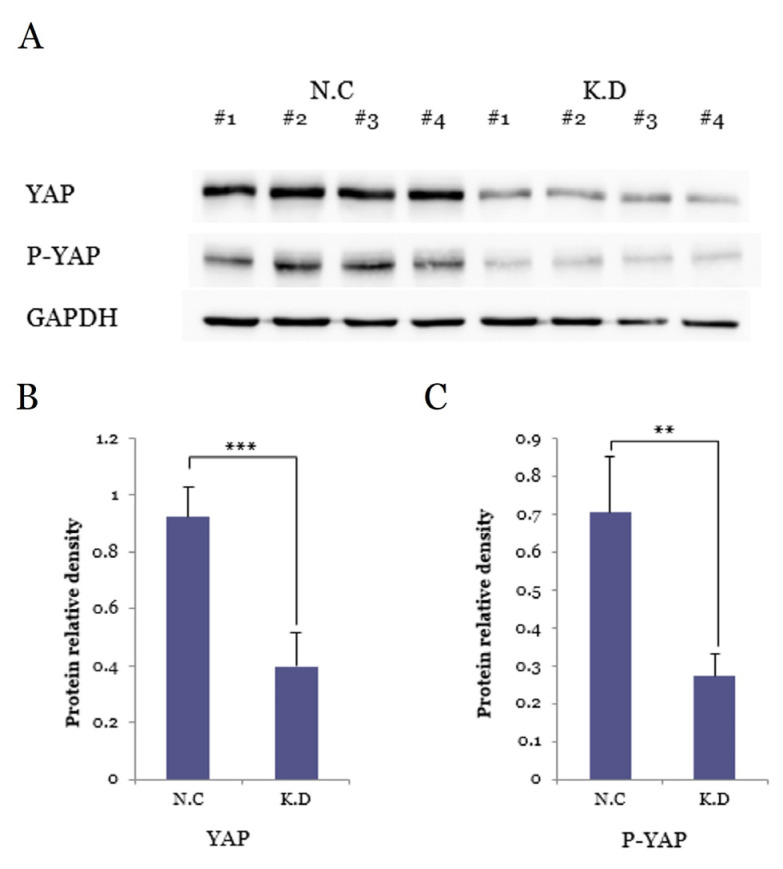
Western blot validation of YAP knockdown in Caski cervical cancer cells. (**A**) Representative blots from four independent experiments showing reduced YAP and P-YAP expression in the knockdown (K.D) group compared with the negative control (N.C), with GAPDH as the loading control. (**B**) Densitometric analysis demonstrating an approximately 50% decrease in YAP levels in the K.D group (*** *p* < 0.001). (**C**) Densitometric analysis showing an approximately 45% reduction in P-YAP levels in the K.D group (** *p* < 0.01). Data represent mean ± SD from four experiments.

Western blot analysis demonstrated significant suppression of both YAP and phosphorylated YAP (P-YAP) in the siRNA-treated knockdown (K.D) group compared with the negative control (N.C) (Figure 3). Densitometric analysis confirmed an approximate 50% reduction in YAP (*** *p* < 0.001) and a 45% decrease in P-YAP (** *p* < 0.01). GAPDH levels remained largely stable, supporting consistent protein loading. These findings verify effective inhibition of YAP signaling at the protein level and provide a solid basis for subsequent downstream analyses.

#### 3.5.3. Functional Consequences of YAP Knockdown on Cell Migration

**Figure 4:**
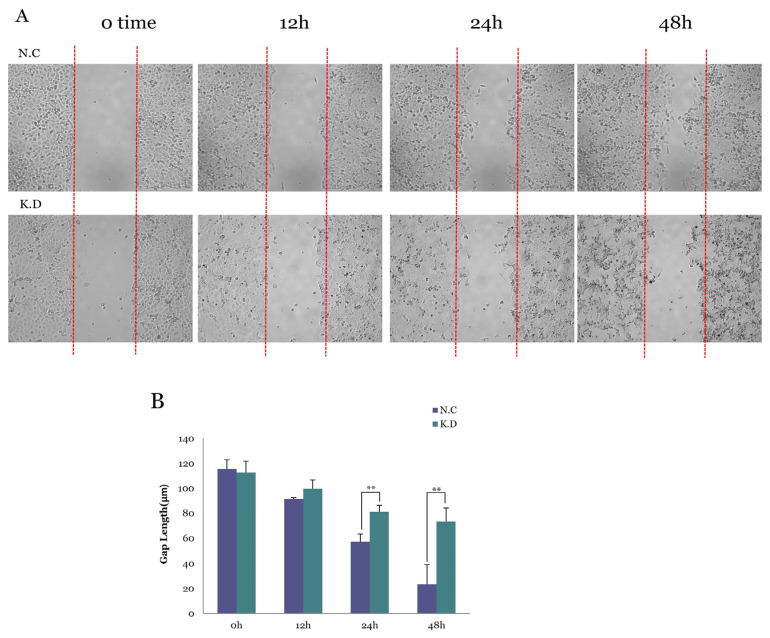
Wound-healing assay demonstrating the effect of YAP knockdown on cell migration in Caski cervical cancer cells. (**A**) Representative wound-healing images of negative control (N.C) and YAP-silenced (K.D) cells captured at 0, 12, 24, and 48 h after scratch formation. Red dashed lines indicate the original wound boundaries. YAP-silenced cells demonstrated visibly slower wound closure compared with N.C cells. (**B**) Quantitative measurement of wound gap distance using ImageJ. Gap width was measured across three independent experiments, and values are presented as mean ± SD. Significant differences between groups were observed at 24 h and 48 h (*p* < 0.01), indicating impaired migratory ability following YAP suppression.

Wound-healing assays were performed over 48 h to evaluate whether YAP silencing affected the migratory behavior of Caski cells (Figure 4). Compared with N.C cells, YAP-deficient cells exhibited markedly slower wound closure, with wider residual gaps observed at 24 and 48 h. These findings indicate that YAP knockdown significantly impairs migration, supporting the role of YAP activation in promoting motility and contributing to the invasive phenotype of cervical carcinoma.

The combined findings from PCR, Western blot, and wound-healing assays indicate that YAP functions as a key regulator of tumor aggressiveness in cervical cancer. Clinically, elevated YAP and p-YAP expression correlated with larger tumor size and more advanced FIGO stage, reflecting enhanced proliferative and invasive potential. Functionally, siRNA-mediated YAP suppression attenuated these aggressive phenotypes in vitro, further supporting its role as a critical driver of cervical cancer progression.

## 4. Discussion

Previous studies have demonstrated that the Hippo pathway is closely associated with human cancer development through its role in regulating tissue growth, including lung, colon, liver, breast, and cervical cancers [16,17,18,19,20,21,30]. In cervical cancer, where HPV significantly contributes to pathogenesis [31], Recent studies indicate that HPV oncoproteins may directly influence Hippo–YAP signaling. HPV E6/E7 have been shown to enhance YAP/TAZ activity through interactions with upstream Hippo regulators and EGFR-related pathways, contributing to cervical cancer progression. In addition, YAP and TAZ appear to exert HPV-type–dependent effects, suggesting that viral genotype may modulate Hippo pathway activation. These findings provide mechanistic support for the relevance of YAP dysregulation in HPV-associated cervical carcinogenesis [19,26,32]. For example, compared with healthy controls, HPV-positive cervical cancer cell lines have exhibited MST1 downregulation, and TAZ dysregulation has been described in an HPV type-dependent manner. Given this evidence, the Hippo pathway has recently emerged as a promising target for cancer therapy.

The FIGO staging system for cervical cancer underscores the significance of tumor size, introducing 2 cm in maximal dimension as a key cutoff for visible cervical cancer [33]. Considering the role of the Hippo pathway in cell fate regulation through YAP/TAZ-mediated TEAD transcriptional activity, it is plausible that Hippo pathway dysregulation impacts cervical tumor growth and stage progression. Consistent with this finding, our study revealed that YAP and p-YAP overexpression was significantly associated with larger tumor size and higher FIGO stage, whereas MST1 and LATS1 showed no such association. Notably, YAP and p-YAP overexpression tended to occur in older patients; however, this did not reach statistical significance. As cervical cancer is frequently diagnosed at an older age compared with endometrial cancer [34], the interplay between Hippo pathway dysregulation and patient age warrants further investigation. However, comprehensive analysis of the association between high-risk HPV infection and Hippo pathway expression was limited owing to the large proportion of missing HPV data in our cohort. Other established prognostic factors, including serum SCC antigen levels [35] and parity, did not significantly differ by Hippo pathway protein expression.

Our results agree with our previous investigation of Hippo pathway expression in early stage endometrioid endometrial cancer [21], where YAP and p-YAP showed an association with tumor size and age. In the present study, cervical cancers larger than 2 cm demonstrated significantly higher odds of YAP and p-YAP overexpression, and stage IB2 or higher, which directly reflects tumor size, revealed comparable findings.

In mammals, the Hippo pathway comprises a phosphorylation-mediated kinase cascade, with MST1/2 and LATS1/2 as central regulators. YAP and TAZ, homologs of Yorkie in Drosophila [36], are the major effectors. YAP/TAZ are phosphorylated when the pathway is “on,” causing their cytoplasmic retention via 14-3-3 binding or proteasomal degradation, thereby inhibiting nuclear translocation and TEAD-driven gene transcription. This event suppresses cell proliferation and tissue overgrowth. Conversely, when Hippo signaling is “off,” dephosphorylated YAP/TAZ enter the nucleus, bind TEAD transcription factors, and promote the transcription of certain genes (CTGF, CYR61, and AXL), which drive proliferation, survival, and angiogenesis. Notably, vestigial-like proteins can compete with YAP/TAZ for TEAD binding and thus serve as YAP/TAZ target gene transcriptional repressors [37,38]. Therefore, the Hippo–TEAD axis represents a crucial hub for tissue growth, gaining increasing attention as a therapeutic target in oncology [16,18,39,40].

Collectively, our main finding that YAP and p-YAP overexpression is correlated with larger tumor size and higher FIGO stage in cervical cancer is consistent with the known biology of Hippo signaling. Since its discovery in 1995, the Hippo pathway has been recognized as a highly conserved cell proliferation, organ size, and tissue homeostasis regulator. Dysregulation of this pathway contributes to oncogenesis across various human cancers, and efforts to exploit Hippo signaling as a therapeutic strategy are underway. Previous studies have further supported this association. Patterson et al. demonstrated that TAZ is upregulated by HPV18 and drives oncogenesis in HPV18-positive cervical cancer cell lines [19]. Wang et al. (2024) reported that Hippo signaling is implicated in cisplatin resistance in cervical cancer [22]. Other studies have offered experimental evidence for Hippo pathway involvement in malignant progression, angiogenesis, and therapeutic resistance in cervical cancer. TEAD inhibitors and other modulators of this pathway are under development; however, their clinical efficacy still poses challenges.

An important consideration in interpreting our findings is the subcellular localization of YAP and p-YAP. Previous studies have demonstrated that the biological effects of YAP are highly context-dependent, with cytoplasmic YAP overexpression associated with lymph node metastasis and recurrence in cervical squamous cell carcinoma [41], whereas nuclear YAP has been shown to enhance oncogenic behavior and modulate radiation sensitivity in endometrial cancer [42]. In our cohort, YAP and p-YAP staining patterns were predominantly cytoplasmic, and only a small subset of tumors exhibited focal or weak nuclear positivity. Because nuclear expression was infrequent and quantitatively limited, a reliable nuclear–cytoplasmic comparison could not be performed, and overall staining intensity was used as the primary semiquantitative measure. This methodological limitation should be considered when interpreting the functional significance of YAP expression patterns. Further studies incorporating digital image analysis or explicit nuclear/cytoplasmic compartment scoring will be required to clarify how differential YAP localization contributes to cervical cancer progression.

In this study, YAP overexpression was observed despite the absence of corresponding changes in the upstream Hippo kinases MST1 and LATS1, and TAZ immunostaining was uniformly negative. This pattern suggests that YAP activation in cervical cancer may occur through non-canonical mechanisms such as integrin-mediated mechano-transduction, GPCR–RhoA signaling, or cytoskeletal tension rather than through canonical MST1–LATS1 suppression [43,44]. Because the lack of TAZ staining may reflect technical limitations, definitive interpretation of TAZ biology is not possible. Overall, these findings support the involvement of alternative regulatory pathways in YAP dysregulation and highlight the need for further mechanistic studies.

The consistent YAP and p-YAP overexpression in cervical cancer, which is correlated with larger tumor size and higher FIGO stage, demonstrates the clinical relevance of Hippo pathway activation in tumor progression. Although p-YAP is traditionally regarded as the inactive, cytoplasmic form generated through LATS-mediated phosphorylation, several studies have shown that elevated p-YAP expression can still correlate with aggressive biological behavior in certain malignancies [45,46]. This phenomenon is thought to reflect broader dysregulation or compensatory activation within the Hippo pathway, rather than simple inactivation of YAP. Indeed, phosphorylated YAP can accumulate in tumors experiencing upstream signaling stress, and a pool of non-phosphorylated, active YAP may coexist even when total p-YAP levels are high. In this context, the increased p-YAP expression observed in our cohort is interpreted as an indicator of pathway imbalance rather than direct YAP activation. Therefore, the association between p-YAP and adverse clinicopathologic features likely reflects altered Hippo signaling dynamics rather than enhanced YAP-dependent transcriptional activity. Our in vitro functional studies have further substantiated these clinical observations. siRNA-mediated YAP knockdown in Caski cervical cancer cells significantly reduced both mRNA and protein levels, confirming effective pathway inhibition. Notably, YAP suppression significantly impaired cellular migration in wound-healing assays, demonstrating the pivotal role of YAP in promoting motility and potentially invasive behavior. These results suggest that YAP activation improves the proliferative capacity and migratory phenotype of cervical cancer cells, which are biological mechanisms consistent with the aggressive clinicopathologic features observed in YAP/p-YAP–overexpressing tumors. Overall, these findings suggest that Hippo pathway dysregulation may play a role in cervical cancer progression through molecular and functional mechanisms, indicating that YAP could serve as a biologically relevant marker and a potential therapeutic target. However, confirmation of its prognostic significance will require studies incorporating survival outcomes.

The strengths of this study encompass the direct evaluation of Hippo pathway core protein expression and its correlation with clinicopathologic features in a relatively large and well-characterized cohort of patients with cervical cancer. Integrating in vitro functional assays further supports the clinical observations, as YAP knockdown experiments demonstrated consistent effects on mRNA, protein expression, and cellular migration. By associating molecular mechanisms with phenotypic outcomes, these combined approaches strengthen the translational relevance of the findings.

However, this study had several limitations. First, the retrospective single-center design and modest sample size may limit generalizability. Second, a substantial proportion (~60%) of cases lacked HPV genotyping and other key clinical data because many patients were referred from outside hospitals without complete records. Given that persistent high-risk HPV infection is the primary etiologic driver of cervical cancer, the absence of viral information limits our ability to determine whether Hippo–YAP pathway alterations occur independently of, or are modulated by, HPV status or genotype. This missing data restricts the interpretation of HPV-specific biological interactions and necessitates a cautious view of the clinical applicability of our findings. Accordingly, the results should be regarded as reflecting Hippo pathway activity within an established cervical cancer cohort rather than elucidating its role in early carcinogenic progression or direct interactions with HPV oncoproteins. Third, this cohort reflects patients treated at a tertiary referral center and therefore may not fully represent nationwide cervical cancer patterns in Korea, it provides an appropriate exploratory dataset for evaluating Hippo pathway–related biomarkers. Forth, although YAP and p-YAP revealed significant associations with tumor size and FIGO stage, key clinical factors, including HPV status, serum SCC antigen levels, and parity, were not significantly correlated, partly owing to incomplete data availability. Fifth, TAZ expression was undetectable in this cohort, likely reflecting technical variability in antibody sensitivity or antigen retrieval conditions. Finally, survival and recurrence outcomes were not analyzed, precluding evaluation of the prognostic implications of Hippo pathway activation. To validate and expand upon these findings, future multicenter studies incorporating molecular profiling, survival analysis, and treatment response data are required.

## 5. Conclusions

This study demonstrated that YAP and p-YAP overexpression in cervical cancer is significantly associated with larger tumor size and higher FIGO stage, highlighting the clinical relevance of Hippo pathway dysregulation in tumor progression. Functional experiments using siRNA-mediated YAP knockdown in Caski cervical cancer cells confirmed that suppressing YAP expression reduces mRNA and protein levels and significantly impairs cell migration. These molecular and functional findings align with the aggressive clinicopathologic features observed in YAP/p-YAP–overexpressing tumors. Collectively, our results indicate that Hippo–YAP signaling contributes to cervical cancer progression through transcriptional and phenotypic mechanisms. Therefore, YAP likely serves as a promising prognostic biomarker and a potential therapeutic target for future molecularly directed therapeutic approaches. To validate these findings, further large-scale studies incorporating HPV status, survival outcomes, and treatment response are warranted.

## Figures and Tables

**Table 1:** Clinicopathologic characteristics of patients with cervical cancer (*n* = 99).

Variable		Value
Age, years (mean [range])		52.6 [26–82]
<50 years, *n* (%) ≥50 years, *n* (%)		45 (45.5%)
		54 (54.5%)
High-risk HPV, *n* (%)	No	5 (5.1%)
	Yes (16 18 31 33 51 52 56 58)	36 (36.3%)
	Non-informative	58 (58.6%)
High parity (≥3), *n* (%)	No	60 (60.6%)
	Yes	39 (39.4%)
SCC antigen (mean [range])		4.73 [0.5–40.8]
<3.0 ng/mL		64 (64.6)
≥3.0 ng/mL		35 (35.4)
Pap smear	Negative	2 (2.0%)
	ASC-US	16 (16.2%)
	LSIL	2 (2.0%)
	HSIL	32 (32.3%)
	SCC	18 (18.2%)
	Non-informative	29 (29.3%)
FIGO stage, *n* (%)	IA1	5 (5.1%)
	IA2	11 (11.1%)
	IB1	18 (18.2%)
	IB2	21 (21.2%)
	IB3	11 (11.1%)
	IIA1	10 (10.1%)
	IIIA2	1 (1.0%)
	IIIC1	19 (19.2%)
	IVB	2 (2.0%)
Tumor size, *n* (%)	<2 cm	32 (32.3%)
	2≥, <4 cm	41 (41.4%)
	≥4 cm	26 (26.3%)
LVSI, *n* (%)	Negative	51 (51.5%)
	Positive	27 (27.3%)
	Non-informative	21 (21.2%)
Stromal invasion, *n* (%)	<1/2	58 (58.6%)
	≥1/2	41 (41.4%)
Surgical approaches, *n* (%)	Laparotomy	27 (27.3%)
	Laparoscopy	72 (72.7%)
Surgical type, *n* (%)	Modified radical	63 (63.6%)
	Radical	36 (36.4%)

HPV, human papillomavirus; SCC, squamous cell carcinoma; Pap, Papanicolaou; ASC-US, atypical squamous cell of undetermined significance; FIGO, International Federation of Gynecology and Obstetrics; LVSI, lymph vascular surface invasion.

**Table 2:** Immunohistochemical staining results of Hippo pathway core proteins in cervical cancer.

Signal Intensity	YAP	p-YAP	MST1	LATS1
Negative	25 (25.3%)	34 (34.3%)	15 (15.2%)	76 (76.8%)
Mild	64 (64.7%)	34 (34.3%)	54 (54.5%)	14 (14.1%)
Moderate	7 (7.1%)	24 (24.3%)	26 (26.3%)	7 (7.1%)
Strong	0 (0%)	4 (4.0%)	2 (2.0%)	1 (1.0%)
Non-informative	3 (3.0%)	3 (3.0%)	2 (2.0%)	1 (1.0%)

YAP, Yes-associated protein 1; p-YAP, phosphorylated Yes-associated protein 1; MST1, mammalian sterile-20-like kinase-1; LATS1, large tumor suppressor kinase-1.

**Table 3:** Association between clinicopathologic features and YAP, p-YAP, and LATS1 expression.

Clinicopathologic Features		YAP Expression			p-YAP Expression			LATS1 Expression		
		Negative (*n* = 25)	Positive (*n* = 71)	*p*-Value	Negative (*n* = 34)	Positive (*n* = 62)	*p*-Value	Negative (*n* = 76)	Positive (*n* = 22)	*p*-Value
Age (years)	<50	8 (32.0%)	36 (50.7%)	0.161	14 (41.2%)	29 (46.8%)	0.671	37 (48.7%)	8 (36.4%)	0.341
	≥50	17 (68.0%)	35 (49.3%)		20 (58.8%)	33 (53.2%)		39 (51.3%)	14 (63.6%)	
High-risk HPV	No	1 (80.0%)	4 (60.6%)	>0.99	1 (70.6%)	4 (61.3%)	0.384	49 (64.5%)	14 (63.6%)	>0.99
	Yes	5 (20.0%)	28 (39.4%)		10 (29.4%)	24 (38.7%)		27 (35.5%)	8 (36.4%)	
High SCC antigen (ng/mL)	<3.0	15 (60.0%)	47 (66.2%)	0.631	18 (52.9%)	43 (69.4%)	0.125	50 (65.8%)	13 (59.1%)	0.618
	≥3.0	10 (40.0%)	24 (33.8%)		16 (47.1%)	19 (30.6%)		26 (34.2%)	9 (40.9%)	
High parity	<3	14 (56.0%)	44 (62.0%)	0.640	18 (52.9%)	40 (64.5%)	0.284	47 (61.8%)	12 (54.5%)	0.623
	≥3	11 (44.0%)	27 (38.0%)		16 (47.1%)	22 (35.5%)		29 (38.2%)	10 (45.5%)	
FIGO	≤IB1	3 (12.0%)	30 (42.3%)	0.007	0 (0%)	29 (46.8%)	<0.001	23 (30.3%)	10 (45.5%)	0.207
	≥1B2	22 (88.0%)	41 (57.7%)		34 (100%)	33 (53.2%)		53 (69.7%)	12 (54.5%)	
Stromal invasion > 1/2	No	14 (56.0%)	43 (60.6%)	0.814	15 (44.1%)	41 (66.1%)	0.051	45 (59.2%)	12 (54.5%)	0.807
	Yes	11 (44.0%)	28 (39.4%)		19 (55.9%)	21 (33.9%)		31 (40.8%)	10 (45.5%)	
Tumor size (cm)	<2	3 (12.0%)	28 (39.4%)	0.013	5 (14.7%)	25 (40.3%)	0.011	23 (30.3%)	9 (40.9%)	0.440
	≥2	22 (88.0%)	43 (60.6%)		29 (85.3%)	37 (59.7%)		53 (69.7%)	13 (59.1%)	

YAP, Yes-associated protein 1; p-YAP, phosphorylated Yes-associated protein 1; LATS1, large tumor suppressor kinase-1; SCC, squamous cell carcinoma; FIGO, International Federation of Gynecology and Obstetrics; LVSI, lymph vascular surface invasion.

**Table 4:** Association between YAP and p-YAP expression with other core proteins of the Hippo pathway.

		YAP				p-YAP			
		Negative	Positive	*p*	Univariable OR (95% CI)	Negative	Positive	*p*	Univariable OR (95% CI)
p-YAP	Negative	15	19	0.003	4.342 (1.695–11.614)	-			
	Positive	10	55						
MST1	Negative	5	10 (77.8%)	0.520	1.6 (0.454–5.085)	4	11	0.569	0.654 (0.169–2.102)
	Positive	20	64 (22.2%)			30	54		
LAST1	Negative	22	54 (80.5%)	0.172	2.716 (0.823–12.348)	28	48	0.454	1.652 (0.607–5.021)
	Positive	3	20 (19.5%)			6	17		
YAP	Negative	-				15	10	0.003	4.342 (1.695–11.614)
	Positive					19	55		

YAP, Yes-associated protein 1; p-YAP, phosphorylated Yes-associated protein 1; MST1, mammalian sterile-20-like kinase-1; LATS1, large tumor suppressor kinase-1; OR, odds ratio; CI, confidence interval.

## Data Availability

The datasets used and/or analyzed in the current study are available from the corresponding author upon reasonable request.

## Supplementary Materials

The following supporting information can be downloaded at https://www.mdpi.com/article/10.3390/medicina61122134/s1: Figure S1, uncropped RT-PCR gels for YAP knockdown; Table S1, siRNA and primer details; Figure S2, Coomassie Blue/Ponceau S staining and raw Western blot data; Figure S3, uncropped Western blots for YAP, p-YAP, and GAPDH.
