# Supplementary material for: Expression of Core Hippo Pathway Proteins in Cervical Cancer and Their Association with Clinicopathologic Parameters
Source: Medicina (Kaunas). 2025 Nov 29;61(12):2134. doi: 10.3390/medicina61122134 (PMC12734526; doi:10.3390/medicina61122134)
Supplement: Supplementary file 1 [file medicina-61-02134-s001.zip › medicina-3978546-supplementary.pdf]

Supplementary Materials for
"Expression of Hippo Pathway Components and Functional Significance of YAP Signaling in Cervical Squamous Cell Carcinoma"

Authors: Jong-Chul Baek, *et al.*

Affiliation: Department of Obstetrics and Gynecology, Gyeongsang National University Hospital, Jinju, Republic of Korea

Corresponding Author: Jong-Chul Baek, M.D., Ph.D. Email: gmfather@gmail.com

Contents

Supplementary Figure S1.
Full-length, uncropped RT-PCR gel images for YAP knockdown experiments corresponding to Figure 2A.

Supplementary Table S1.
siRNA and primer information, including catalog numbers, sequences (when available), and vendor details.

Supplementary Figure S2.
Coomassie Blue and Ponceau S staining images demonstrating total protein loading and membrane transfer quality, with raw Western blot data for YAP, p-YAP, and GAPDH.

Supplementary Figure S3.
Uncropped Western blot images for total YAP, phosphorylated YAP (p-YAP), and GAPDH corresponding to Figure 3A.

General Description

The supplementary materials provide full experimental transparency by including uncropped molecular data, loading controls, and technical details required for reproducibility. All supplemental figures directly correspond to the results presented in the main manuscript and allow independent verification of the experimental workflow. Supplementary Materials: Additional figures and data supporting the findings of this study are provided in the Supplementary File (Figures S1–S3, Table S1).

Supplementary Figure S1: Full-length, uncropped RT-PCR gel images for YAP knockdown experiments corresponding to Figure 2A. Includes four independent experiments (#1–#4) with complete gel fields used for densitometric quantification.

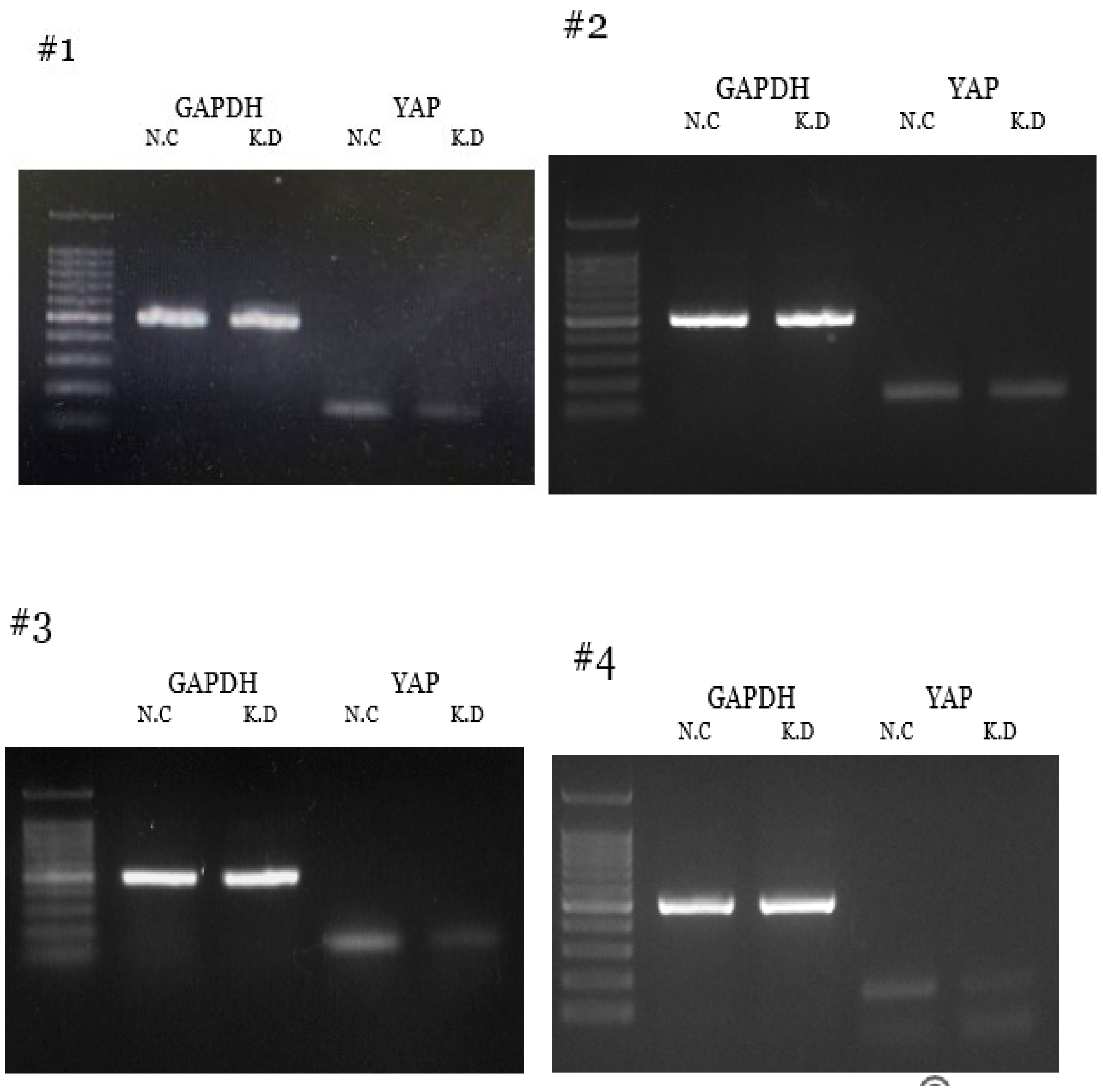

Supplementary Table S1: Detailed information on the siRNAs and primers used in this study, including catalog numbers, sequences (when available), and vendor specifications. This table provides essential methodological details for experimental replication.

Category	Target	Manufacturer	Catalog No.	Sequence Information
**siRNA**	YAP1	Bioneer	10413-2	**Sense:** AGA ACC GUU UCC CAG ACU A–tt **Antisense:** UAG UCU GGG AAA CGG UUC U–tt
**siRNA**	Negative control	Bioneer	SN-1002	Sequence not disclosed by manufacturer
**Primer (RT-PCR)**	YAP1	Bioneer	P245469	Sequence not provided by manufacturer
**Primer (RT-PCR)**	GAPDH	Cosmogenetech	—	**Forward:** 5′-GTC CAC CAC CCT GTT GCT GTA G-3′ **Reverse:** 5′-CAA GGT CAT CCA TGA CAA CTT TG-3′

Supplementary Figure S2.

Coomassie Blue and Ponceau S staining images confirming total protein loading and uniform transfer quality for Western blot membranes. Includes raw, unprocessed band images for YAP, p-YAP, and GAPDH used in Figure 3A.

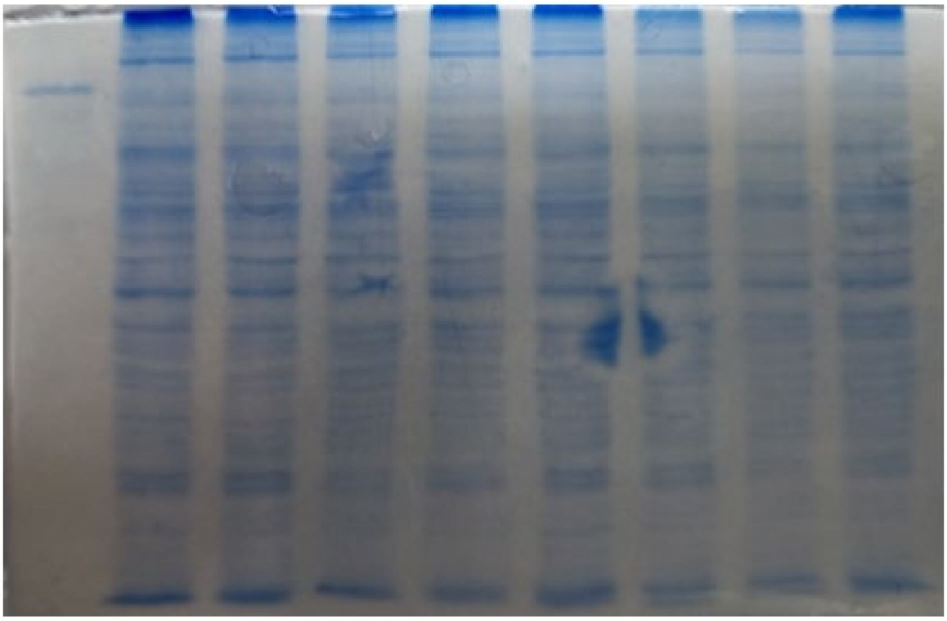

Coomassie blue

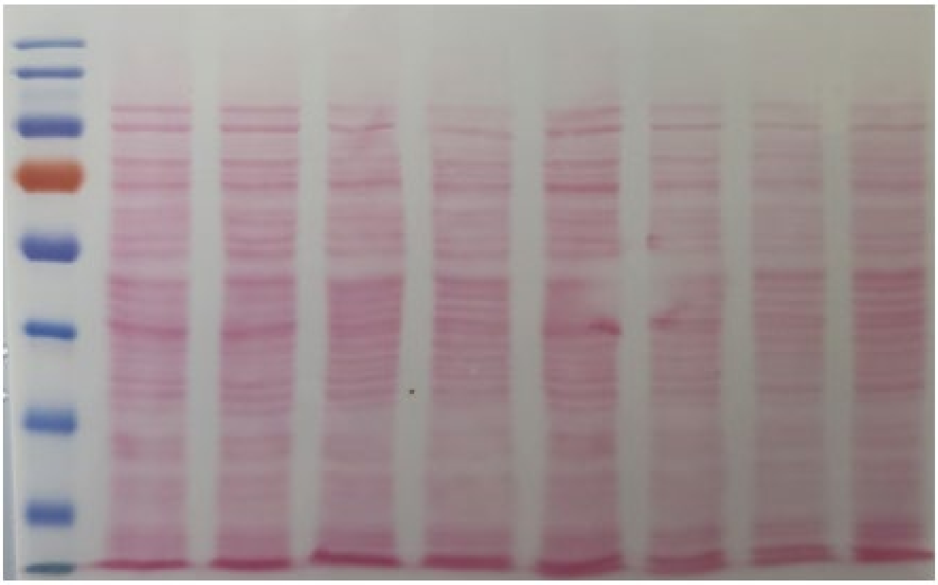

Ponceau

Supplementary Figure S3: Uncropped Western blot images for total YAP, phosphorylated YAP (p-YAP), and GAPDH corresponding to Figure 3A. Displays the original blot images used for quantification.

YAP raw date

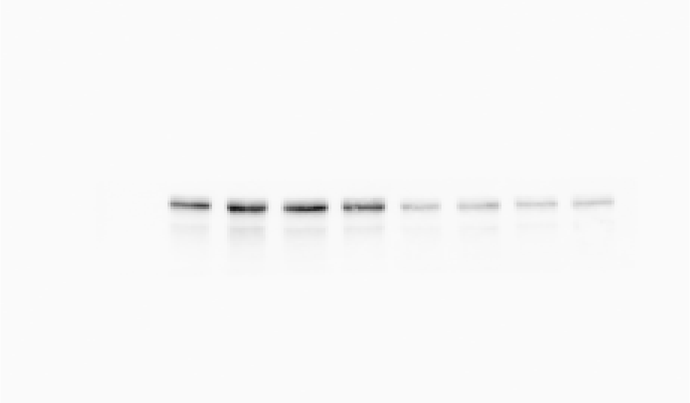

P-YAP raw date

GAPDH raw date
